# Peace, a MYB-like transcription factor, regulates petal pigmentation in flowering peach ‘Genpei’ bearing variegated and fully pigmented flowers

**DOI:** 10.1093/jxb/ert456

**Published:** 2014-01-22

**Authors:** Chiyomi Uematsu, Hironori Katayama, Izumi Makino, Azusa Inagaki, Osamu Arakawa, Cathie Martin

**Affiliations:** ^1^Botanical Gardens, Graduate School of Science, Osaka City University, Osaka 576-0004, Japan; ^2^Food Resources Research and Education Center, Kobe University, Hyogo 675-2103, Japan; ^3^Faculty of Agriculture and Life Science, Hirosaki University, Aomori 036-8561, Japan; ^4^Department of Metabolic Biology, John Innes Centre, Norwich NR4 7UH, UK

**Keywords:** Anthocyanin, flowering peach, MYB-like transcription factor, *Peace*, *Prunus persica*, variegation.

## Abstract

Flowering peach *Prunus persica* cv. Genpei bears pink and variegated flowers on a single tree. The structural genes involved in anthocyanin biosynthesis were expressed strongly in pink petals but only very weakly or not at all in variegated petals. A cDNA clone encoding a *MYB*-like gene, isolated from pink petals was strongly expressed only in pink petals. Introduction of this gene, via biolistics gave magenta spots in the white areas of variegated petals, therefore this gene was named as *Peace* (*pe*ach *a*nthocyanin *c*olour *e*nhancement). Differences in *Peace* expression determine the pattern of flower colouration in flowering peach. The R2R3 DNA-binding domain of Peace is similar to those of other plant MYBs regulating anthocyanin biosynthesis. Key amino acids for tertiary structure and the motif for interaction with bHLH proteins were conserved in Peace. Phylogenetic analysis indicates that Peace is closely related to AtMYB123 (TT2), which regulates proanthocyanidin biosynthesis in *Arabidopsis*, and to anthocyanin regulators in monocots rather than to regulators in dicots. This is the first report that a TT2-like R2R3 MYB has been shown to regulate anthocyanin biosynthesis.

## Summary

*Peace* expression determined flower colouration whether pigmented or variegated. In spite of the close relationship between Peace and AtMYB123, a proanthocyanidin regulator, Peace regulates anthocyanin biosynthesis in flowering peach.

## Introduction

Pigmentation is an important trait for ornamental plants especially flowers. Anthocyanins, one of the major pigments in the flower kingdom, are widely distributed among higher plants. Six basic types of anthocyanidin are found in plants (pelargonidin, cyanidin, delphinidin, peonidin, petunidin, and malvidin) which provide different colours in flowers of different species depending on the degree of side-chain decoration, (glycosylation and acylation) (Glover and Martin, 2012). Horticulturalists, breeders, and geneticists have been eager to produce unique cultivars with attractive flower colour traits (Forkmann, 1993; Martin and Gerats, 1993; Holton and Cornish, 1995).

There are two categories of genes involved in the biosynthesis of anthocyanins (Martin *et al.*, 1991; Jackson *et al.*, 1992). The first category includes structural genes encoding enzymes catalysing each step of the biosynthetic pathway such as chalcone synthase (CHS), chalcone isomerase (CHI), flavanone 3-hydroxylase (F3H), dihydroflavonol 4-reductase (DFR), anthocyanidin synthase (ANS), and UDP glucose-flavonoid 3-*O*-glucosyltransferase (UFGT). A second category includes regulatory genes encoding MYB-like transcription factors (Sablowski *et al.*, 1994; Martin and Paz-Ares, 1997; Kranz *et al.*, 1998; Stracke *et al.*, 2001), basic helix-loop-helix (bHLH) transcription factors (Bailey *et al.*, 2003; Heim *et al.*, 2003; Toledo-Ortiz *et al.*, 2003), and WD repeat proteins (de Vetten *et al.*, 1997; Walker *et al.*, 1999) which regulate the expression of the structural genes co-ordinately (Quattrocchio *et al.*, 1993; Baudry *et al.*, 2004; Zimmermann *et al.*, 2004).

Flowering peach, *Prunus persica* (L.) Batsch. cv. Genpei is one of the ornamental peach cultivars which bears fully pigmented pink flowers and variegated flowers on a single tree. The cultivar name ‘Genpei’ has its origin in Japanese history, therefore it is iconic in Japanese culture with a colour image of red and white. Pink flowers and variegated flowers of Genpei are believed to result from mutation as opposed to a graft chimera. Branches with pink flowers reproducibly bear only pink flowers every year, but branches with variegated flowers occasionally produce fully pigmented flowers. This phenomenon is similar to the unstable variegation caused by insertion and excision of class II transposable elements as observed in snapdragon (Harrison and Carpenter, 1979; Bonas *et al.*, 1984; Martin *et al.*, 1985; Sommer *et al.*, 1985; Upadhyaya *et al.*, 1985; Martin *et al.*, 1988), petunia (Doodeman *et al.*, 1984; Gerats *et al.*, 1990; Snowden and Napoli, 1998; van Houwelingen *et al.*, 1998), morning glory (Epperson and Clegg, 1987; Inagaki *et al.*, 1994; Habu *et al.*, 1998), and carnation (Itoh *et al.*, 2002; Nishizaki *et al.*, 2011). Chaparro *et al.* (1995) suggested the involvement of an active transposable element as a cause of flower colour variegation in flowering peach cv. Pillar. However, no transposon has yet been detected in Pillar.

Bud mutation has commonly been used as a convenient tool to obtain new cultivars from fruit trees, even though the mechanisms of mutation remain unclear (Imai, 1935; Shamel and Pomeroy, 1936; Whitham and Slobodchikoff, 1981). Flowering peach with variegated flowers could be a good system to reveal the mechanisms of bud mutation in woody species. Generally, fruit trees are genetically heterozygous and have long life cycles, so genetic analysis is problematic. Flowering peach Genpei, has two phenotypes, pink and variegated flowers, within one tree, i.e. two genotypes, wild type and mutant, co-exist within the same tree. These two genotypes probably possess the same genetic background except for the difference in flower colour trait and so are equivalent to isogenic lines.

In some fruit species such as apple, pear, peach, grape, and citrus, many cultivars have been derived from a few cultivars via bud mutation (Shamel and Pomeroy, 1936; Butelli *et al.*, 2012). In Japan, more than two-thirds of peach cultivars are thought to have arisen as chance seedlings or sports by mutation from ‘Hakuto’ or its relatives (Yamamoto *et al.*, 2003a, b). A high frequency of bud mutations in certain cultivars might suggest involvement of a transposable element in their creation. If transposable elements could be found in unstable flowering peach, it might be possible to use them to create new mutations and as tags to isolate the genes responsible for useful traits.

The isolation of the *Peace* gene encoding a Myb transcription factor that regulates petal pigmentation in flowering peach is reported here. *Peace* is expressed strongly only in pink petals and can complement the magenta colour in the white areas of variegated petals when introduced by particle bombardment. Comparison of the deduced amino acid sequence of Peace with other Myb transcription factors, suggests that *Peace* plays a role in inducing the anthocyanin biosynthetic pathway in peach petals.

## Materials and methods

### Plant material

**Fig. 1.**
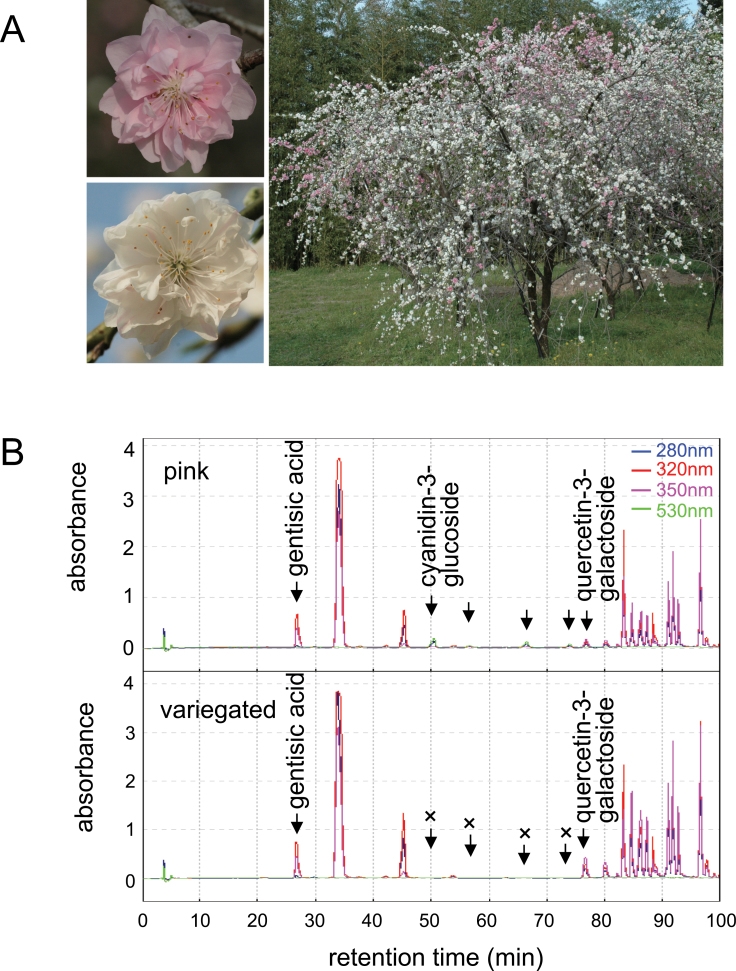
Peach flowers used in this study and pigment analysis. (A) Peach tree ‘Genpei’ bearing fully pink coloured flowers and variegated flowers within a single tree. (B) HPLC profiles obtained from pink petals and variegated petals, respectively. Absorbance spectrum at the wave length of 280, 320, 350, and 530nm are given.

A weeping type tree of flowering peach (*Prunus persica* cv. Genpei, Plant ID No. 4F0199) bearing double flowers maintained at the Botanical Gardens of Osaka City University, Japan, was analysed in this study (Fig. 1A). Fully pigmented pink flowers and variegated flowers were borne simultaneously within one tree. One branch bearing pink flowers (P2 branch) and another branch bearing variegated flowers (V2) were marked to collect flower material.

### HPLC assay for pigment analysis

For the pigment analysis using HPLC, flower petals collected from newly opened flowers were frozen in liquid nitrogen and stored at –80 °C until use. Frozen petals from one flower were placed in the pre-cooled mortar with liquid nitrogen and ground thoroughly using a chilled pestle. Extraction buffer containing 300 μl of 15% acetic acid in methanol and 100 μl of 0.1% gentisic acid was added to the ground material and kept on ice for 1h. The extraction mixture was centrifuged at 10 000rpm for 10min at 4 °C. The supernatant was filtered using a Millipore filter of 0.45 μm and then applied for HPLC analysis using NANOSPACE SI-1 (SHISEIDO, Japan) equipped with Photo Diode Array Detector 2017 (SHISEIDO, Japan) and CAPCELL PAK C18 UG 120 column (4.6 mm in diameter × 150 mm in length). Solvent A (1.5% phosphate solution) and solvent B (1.5% phosphate, 20% formic acid, 25% acetonitrile solution) were used with the following gradients: A; 90–70% (0–30min), 70–65% (30–40min), 65–55% (40–70min), 55–0% (70–90min), and B; 100% after 90min. The flow rate was 100 μl min^–1^ and the detection wavelengths were 280, 320, 350, and 530nm.

In order to identify peaks obtained from HPLC profiles, chlorogenic acid, *p*-coumaric acid, eriodictyol, naringenin, quercetin dihydrate, quercetin-3-galactoside, catechin, epicatechin, cyanidin chloride, and cyanidin-3-*O*-glucoside were used as standards for HPLC analysis, each prepared as a 0.1% solution with 15% acetic acid in methanol. For the quantification of identified peaks, 0.1% gentisic acid prepared with 15% acetic acid in methanol was used as an internal standard.

### DNA extraction and blotting to the membrane

Total DNA was extracted from frozen petals collected separately from pink flowers and variegated flowers just after blooming using a CTAB method (Katayama and Uematsu, 2003). DNA (10 μg) was digested with *Bam*HI, *Dra*I, *Eco*RI or *Hin*dIII and fractionated by 0.8% agarose gel electrophoresis then blotted onto the Hybond-N^+^ (GE Healthcare, UK) nylon membranes.

### Probe preparation and signal detection

Partial genomic clones of the *CHS*, *CHI*, *F3H*, *DFR*, and *AS* genes were obtained from pink flowers by PCR cloning using degenerate primers as follows:

CHS/F2 (5′-CACCCACYTKGATWSYYTIG-3′),

CHS/R1 (5′-GCWATCCAGAABADVGAGTT-3′),

CHI/F1 (5′-SYYAARTGGAARGGYAARAC-3′),

CHI/R3 (5′-DGAHBCRTCTTTGGARAAGC-3′),

F3H/F2 (5′-CTACAACGACTTCAGCAACG-3′),

F3H/R1 (5′-CARTCTTGCACWGCTTCTCC-3′),

DFR/F8 (5′-GCITCIGGITTYATWGGITCATGGC-3′),

DFR/R8 (5′-CGTAGCATSGTGIGAIGARCAAATG-3′),

AS/F2 (5′-CUATCCCIAAAGAGTACATC-3′) and

AS/R1 (5′-GAAAACAGCCCATGAAATCC-3′).

Degenerate primers contained the following nucleotides; B=T+C+G, D=A+T+G, H=A+T+C, K=T+G, R=A+G, S=C+G, V=A+C+G, W=A+T, Y=C+T, and I which indicates deoxyinosine. Primers of Malubi/F (5′-ATGATCGAGGTGGTGCTGAA-3′) and Malubi/R (5′-AGTCCTTGAGGGTGATGTTGG-3′) both designed based on the *Malus* ubiquitin sequence (GenBank DT00167) were used to amplify the ubiquitin gene from flowering peach. Amplified fragments were cloned using a TA cloning kit (Life Technologies, USA). Clones were subjected to sequencing to confirm they had the appropriate insert. Clones of the *CHS*, *CHI*, *F3H*, *DFR*, *ANS*, and *Ubiquitin* genes were used as homologous probes. cDNA clones of *PAL* and *UFGT* obtained from *Antirrhinum majus* (*A. majus*) were also used as a probes. Hybridization was performed with ECL non-radioactive DNA labeling and detection kits (GE Healthcare, UK).

### RNA extraction and first strand cDNA synthesis

Two hundred milligrams of petals were collected separately from pink and variegated flower buds ranging from 6–9mm in diameter. Petals removed from flower buds were immediately frozen in liquid nitrogen and subjected to total RNA extraction using an RNeasy Plant Mini kit (Life Technologies, USA). First strand cDNA was prepared using a cDNA synthesis kit (GE Healthcare, UK) according to the manufacturer’s instructions. On occasion, an adapter primer having dT_17_ (B26) was used (Frohman *et al.*, 1988; Schwinn *et al.*, 2006). Six micrograms of total RNA was used for one reaction and cDNA was diluted into 1ml with sterilized water. 10 μl of first-strand cDNA solution was used for the RT-PCR and 3′ RACE PCR.

### Partial cloning of a peach *MYB*-like gene by 3′ RACE PCR

The 3′ end of the peach *MYB* gene was obtained by 3′ RACE amplification using 10 μl of first-strand cDNA solution as a template with the G1709 primer (forward, 5′-AAAAGCTGCAGACTTAGG TGGTTGAATTATCTAAAGCC-3′) designed for *Ros*1 amplification in snapdragon (Schwinn *et al.*, 2006) and B25 adapter primer (reverse, 5′-GACTCGAGTCGACATCG-3′) (Frohman *et al.*, 1988). PCR amplification was performed using Gene Amp^®^ PCR System 9700 (Life Technologies, USA) with the following thermal conditions; 94 °C for 5min then a repeat of 30 or 40 cycles of 94 °C 100%/94 °C for 48 s, 50 °C 50%/50 °C for 75 s, 72 °C 80%/72 °C for 3min and, finally, 72 °C for 10min for extension. Amplified fragments were cloned using pGEM-T Easy Vector System (Promega, USA).

### Gene expression confirmed by RT-PCR Southern hybridization

Expression of the structural genes was detected by RT-PCR amplification using the first-strand cDNA solution as a template with primers used for probe preparation and the B25 adapter primer described above. For amplification of *MYB*-like genes, the G1709 forward primer was used (Schwinn *et al.*, 2006). PCR amplification was performed using the same conditions as used for cloning the peach *MYB*-like genes. Amplified fragments were fractionated using 1.4% agarose gel electrophoresis then blotted onto a Hybond-N^+^ membrane. Signals were generated according to the methods described for probe preparation and signal detection.

### 5′ RACE PCR

The 5′ end of the peach *MYB* gene was determined by 5′ RACE System (Life Technologies, USA) according to the manufacturer’s instructions using the PGS240 primer (5′-GCTCTTGTTTCTCTTC TTCTTCGTGTTGTCGTTAA-3′) as a GSP1 (Gene-Specific Primer 1) and PGS42 primer (5′-CTCTTCCTCCTGAGTTA TATTTCCCCT TTTGATAT-3′) as a GSP2 (Gene-Specific Primer 2). Amplified product was cloned using the pGEM-T Easy Vector System (Promega, USA) and sequenced.

### Preparation of an expression vector harbouring the peach MYB gene

The PMintF primer (5′-TTGCCAATTCGC CGAAGTTTGGA-3′) and the PMintR primer (5′-AGGTTATGCTTATGGTCAAC ATTAT-3′) were designed based on the sequence information from 3′ RACE and 5′ RACE of the peach MYB gene. First-strand cDNA was amplified using this primer set. PCR product was diluted 100-fold and used for GATEWAY cloning (Life Technologies, USA) according to the manufacturer’s instructions. PCR amplification was performed with primers in which 25bp *att*B1 and 25bp *att*B2 sequences plus four termial Gs were added to the intermediate F and R primers, respectively. The Entry clone was made by recombination between *att*B-PCR product of the peach MYB gene and pDONR 207 using the BP reaction. The peach MYB gene (*Peace* cDNA sequence, GenBank/EMBL/DDBJ accession AB897865) was then transferred to the expression vector of pJAM1500 (a destination vector derived from pJIT60 with a Gateway destination cassette inserted in the *Sma*I site of the polylinker between the double CaMV 35S promoter and the CaMV terminator sequence) using the LR reaction.

### Complementation analysis by particle bombardment

Variegated flower buds (just before opening) were bombarded using a particle inflow helium gun (Vain *et al.*, 1993; Schwinn *et al.*, 2006) with genes encoding the transcription factors: Rosea1, Rosea2, Venos, and Mutabilis from *Antirrhinum majus* or peach *MYB* cDNA cloned into the expression vector. About 2mg of gold particles (size 0.95) were coated with 10 μg of plasmid DNA of each transcription factor and 5 μg of *YFP* gene DNA prepared in 20 μl by adding 100 μl each of the Xho buffer (30 μl of 5M NaCl, 5 μl of 2M TRIS pH 8.0, 965 μl of SDW), 0.1M spermidine, 25% PEG (mol. wt. 1300–1600), and 2.5M CaCl_2_. This mixture was incubated for a few minutes and then, after pulse centrifugation, the supernatant was removed and the pellet was washed with absolute ethanol. Gold particles were resuspended in 2ml of absolute ethanol. Horizontally placed tubing was filled with this gold particle suspension using a syringe and then left for a few minutes to settle the gold particles at the bottom of tubing. A piece of paper was placed at the end of the tubing to drain the ethanol. The tubing was dried completely with nitrogen gas. This dried tubing was then cut and inserted into the cartridge of the gene gun for shooting. Each flower bud was shot three times at 300 psi pressure. After shooting, branches were kept at room temp (about 20 °C). After 24h, petals were removed from the flowers and placed on glass slides. Magenta spots, generated by complementation, were counted under bright field of a dissection fluorescent microscope (LEICA MZFLIII, Germany) and, in the same field, yellow spots generated by expression of the *YFP* gene were counted under dark field.

### Cloning of the *Peace* gene using a genomic library

Cloning of the *Peace* gene (*Peace* genomic sequence, GenBank/EMBL/DDBJ accession AB897866) from genomic DNA was carried out following the directions for the Stratagene phage cloning system (Stratagene, USA). Total DNA extracted from V2 petals was partially digested with *Sau3A*I (TAKARA, Japan). After a partial fill-in reaction, total DNA fragments were ligated with lambda FIX/*Xho*I partial fill-in treated DNA (Stratagene, USA), and a packaging reaction was carried out using GigaPack Gold (Stratagene, USA). Recombinant phages were selected by plaque hybridization with *Peace* cDNA (clone cPpP14) as a probe. Plaque hybridization was carried out using an ECL non-radioactive DNA labelling and detection kit (GE Healthcare, UK).

### Cloning of the PprMYB10 homologue from flowering peach

A partial genomic sequence homologous to the *PprMYB10* (*Prunus persica* MYB10, Lin-Wang *et al.*, 2010) gene was obtained from DNAs of pink petals by PCR cloning followed by sequencing. Three primer sets:

A2F (5′-ATGGAGGGTTATGACTTGAGTGTGAGA-3′) and A2R (5′- TACTTCATCCTCTGCAAACTCTCCTTTC-3′);

B2F (5′-GTGCAGGAAGAGCTGTAGACTAAGG-3′) and B2R (5′-CTCCCACCAATCACGTTGAGTATGG-3′); and

C2F (5′-AAAGACCATAATAAGGCAACAACCAAG-3′) and C2R (5′-GGTCCACGCTAAAAGAGAAATCACC-3′)

were used for amplification. The A2F primer started from the start codon of *PprMYB10*. Three primer sets were designed to overlap each other and cover almost the entire region of the *PprMYB10* gene.

### Phylogenetic analysis

Amino acid sequences of 21 MYB transcription factors including Peace were aligned using CLUSTALW [version 2.1]. The phylogenetic tree was generated using Njplot [version 2.3].

## Results

### Pigment responsible for the pink colour in petals

HPLC analysis revealed that cyanidin-3-glucoside was the major pigment in pink peach flowers (Fig. 1B). Pink flowers contained 62.3 μg of cyanidin-3-glucoside per flower. By contrast, variegated flowers contained only 0.6 μg of cyanidin-3-glucoside per flower, so that the level of anthocyanin in variegated flowers was only 1% of those in pink flowers. Variegated flowers contained higher levels of quercetin-3-galactoside (108.2 μg per flower), which was twice the level of that in pink flowers (56.5 μg). These results suggested that the latter part of the anthocyanin biosynthetic pathway catalysed by DFR, ANS, and UFGT is suppressed in variegated flowers.

### Genomic Southern hybridization

**Fig. 2.**
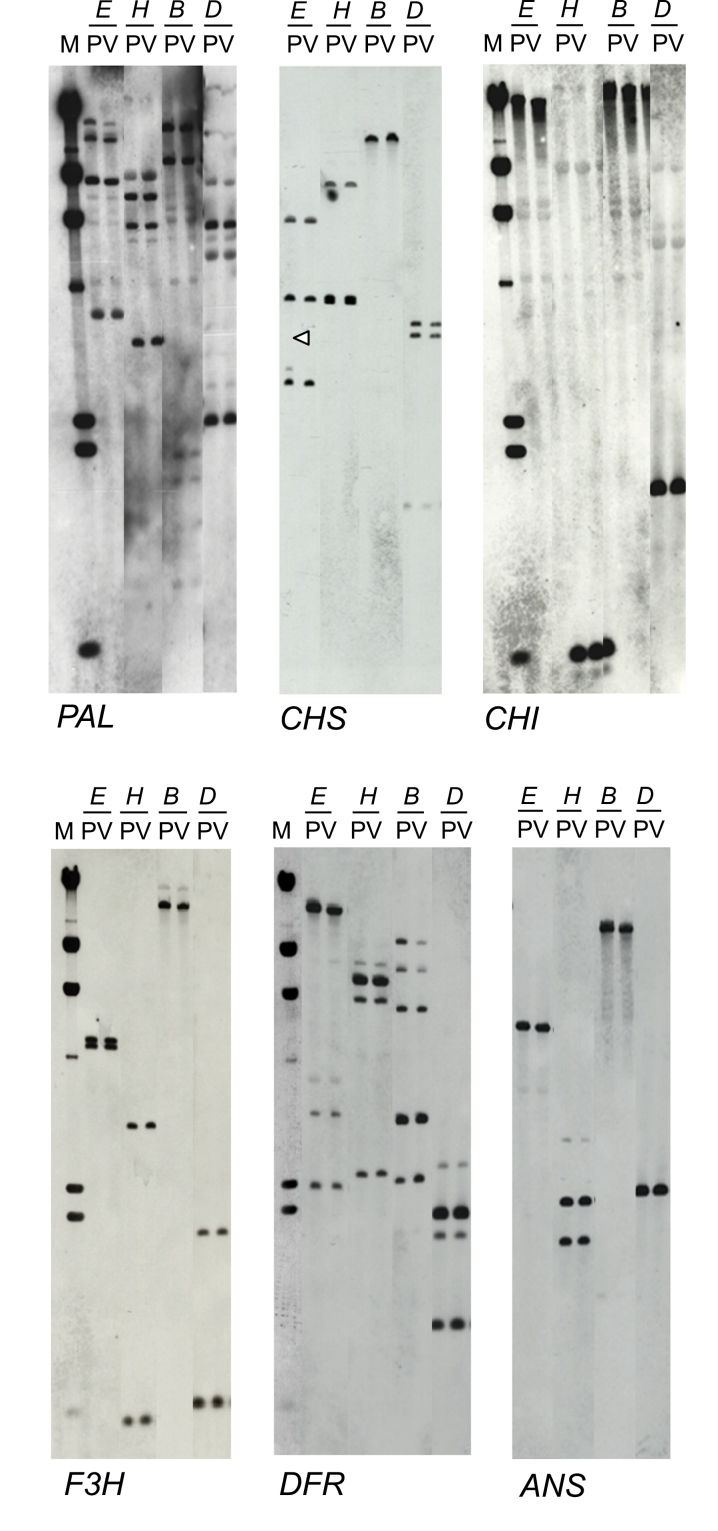
Genomic Southern hybridization probed with *PAL*, *CHS*, *CHI*, *F3H*, *DFR*, and *ANS* genes, respectively. Total DNAs of P, pink petal, and V, variegated petal, were digested with *E*, *Eco*RI; *H*, *Hin*dIII; *B*, *Bam*HI; and *D*, *Dra*I. Only a slight difference indicated by a triangle was seen.

DNA extracted from pink flowers and variegated ones was digested with restriction enzymes, separated by gel electrophoresis, subjected to Southern hybridization and probed with genomic or cDNA clones encoding genes involved in the anthocyanin biosynthetic pathway (Fig. 2). The only polymorphism observed was in the *Eco*RI digestion, for the *CHS* gene. A very weak hybridization signal to a 2.7kb fragment was seen only in DNA from pink petals, but not in DNA from variegated petals. No other polymorphic patterns were observed for other restriction enzymes such as *Bam*HI, *Dra*I, and *Hin*dIII, so it was concluded that it was unlikely that this weak signal was responsible for the difference in flower colour phenotype. Other probes such as *PAL*, *CHI*, *F3H*, *DFR*, *ANS*, and *UFGT* did not show any polymorphisms between pink and variegated petals. No other differences were detected at the DNA level that could explain the different flower colour phenotypes.

### Gene expression in the flower bud

**Fig. 3.**
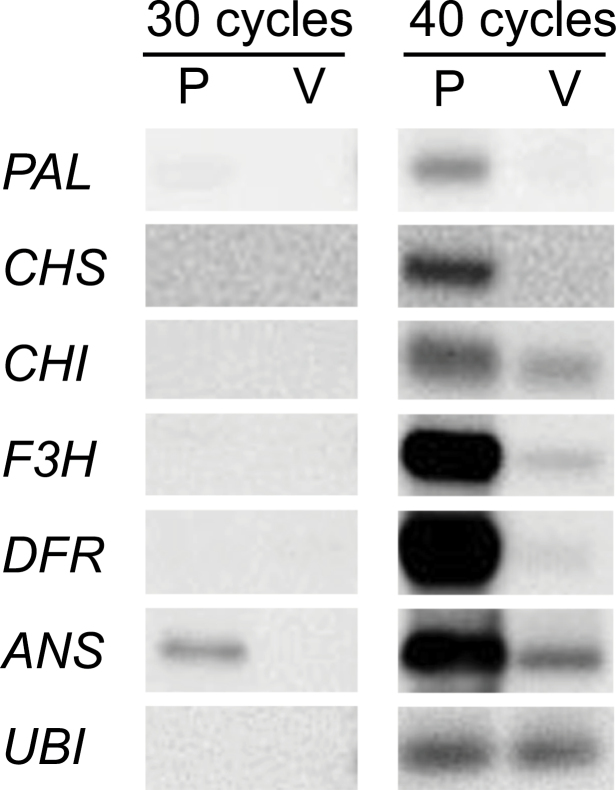
Gene expression in the petals of young flower buds examined by RT-PCR Southern hybridization. P, pink petal; V, variegated petal.

Expression of structural genes, which encode enzymes in the anthocyanin biosynthetic pathway, was analysed by RT-PCR Southern hybridization using RNAs extracted from young petals of pink and variegated flower buds. All genes were very strongly expressed in pink petals, whereas in variegated petals *CHI*, *F3H*, and *ANS* showed very weak expression compared with pink petals (Fig. 3). *PAL*, *CHS*, and *DFR* gene expression in variegated petals was not detected even after 40 cycles of amplification. This result suggested that the white sectors in variegated flowers involved loss of function of a gene regulating the expression of structural genes of the anthocyanin biosynthetic pathway.

### Complementation of pigment biosynthesis

To assess whether heterologous transcription factors could regulate the anthocyanin biosynthetic pathway in flowering peach, four genes encoding transcription factors from *Antirrhinum majus*, i.e. *Rosea1*, *Rosea2*, *Venosa*, and *Delila* (Schwinn *et al.*, 2006) were bombarded into the white area of variegated petals together with the *YFP* gene. All introduced genes were cloned into the pJIT60 vector under the control of the double CaMV 35S promoter. Twenty-four hours later, bombarded petals were removed from the flower and observed under the dissection microscope equipped with a fluorescent light source. Magenta spots were observed when an introduced gene for the transcription factor could complement anthocyanin biosynthesis (Table 1). The same area of petal tissue was observed under fluorescent light to count the number of yellow spots generated by expression of the *YFP* gene. The complementation ratio was calculated from the number of magenta spots divided by the number of yellow spots (Table 1). *Rosea1*, which encodes one of the MYB transcription factors controlling anthocyanin biosynthesis in *A. majus*, showed the highest complementation ratio of 25.3%, whereas *Rosea2* and *Venosa* (which also encode MYB transcription factors; Schwinn *et al.*, 2006) and *Delila* (which encodes a bHLH protein controlling anthocyanin production; Goodrich *et al.*, 1992), showed lower complementation ratios. Therefore, expression of a MYB-like transcription factor, functionally homologous to Rosea1, might be disrupted in the white parts of variegated petals of flowering peach, because cyanidin-3-glucoside was not produced there.

**Table 1.** Magenta spot ratio observed after particle bombardment

Transcription factors	Magenta spot/yellow spot (%)
Negative control	0.0
*YFP* control	0.0
*Rosea1*	25.3
*Rosea2*	9.1
*Venosa*	4.0
*Delila*	7.7

### Isolation of a *MYB*-like gene from pink peach flower buds

**Fig. 4.**
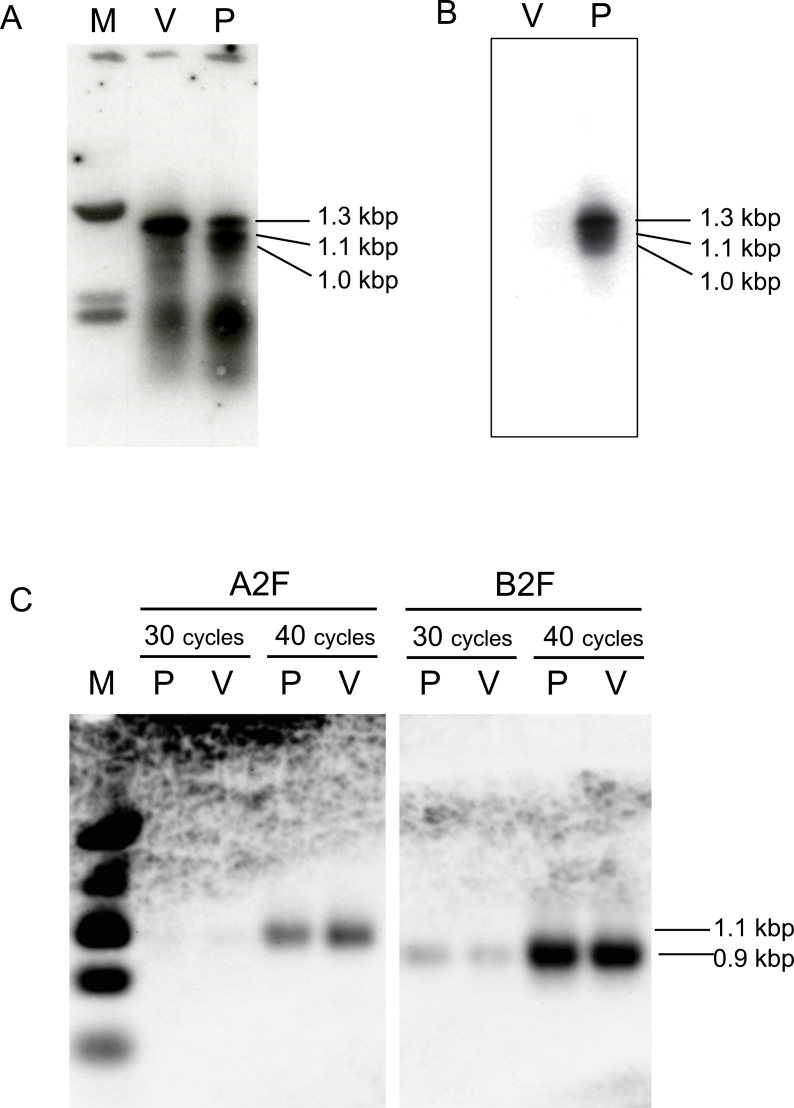
*MYB*-like gene expression in young petals of variegated (V) and pink (P) flower buds. (A) Amplified using the G1709 primer and probed with the *Rosea1* gene of *Antirrhinum majus*. (B) Amplified using the G1709 primer and probed with clone cPpP14 derived from pink flowers of flowering peach. (C) Amplified using the A2F or B2F primer for *PprMYB10* and probed with the 3′ region of the *PprMYB10* gene. Number of cycles indicates the amplification cycle of PCR performed.

To confirm the involvement of a MYB-like transcription factor in controlling flower pigmentation, expression of *MYB*-like genes was examined in pink and variegated petals by RT-PCR Southern hybridization. The G1709 primer, based on the petunia *AN2* and maize *C1* gene sequences (Schwinn *et al.*, 2006) and the B25 primer for 3′ RACE amplification (Frohman *et al.*, 1988) were used. The *Rosea1* gene was used as a probe. cDNA from both pink and variegated petals gave strong signals for a 1.3kb transcript (Fig. 4A). However, there was a difference in cDNAs from pink and variegated petals for bands of 1.0kb and 1.1kb. For both these fragments, pink petals showed stronger expression than variegated petals. Therefore these 1.0kb and 1.1kb transcripts could be candidates for genes regulating the difference in anthocyanin biosynthesis in flowering peach.

**Fig. 5.**
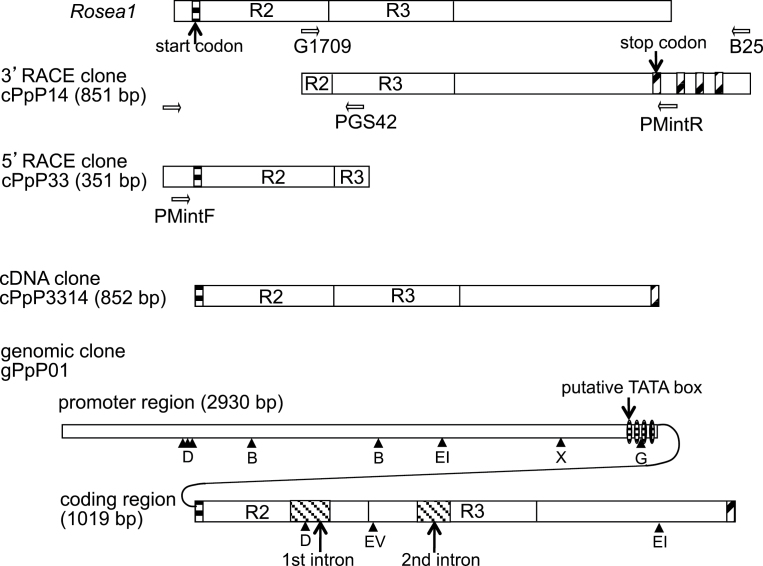
Schematic strategy for the cloning of the peach MYB-like gene based on the sequence information of *Rosea1* and the peach MYB-like gene structure obtained from cDNA and genomic DNA. Restriction enzyme sites used in Southern blots were indicated by a triangle with B for *Bam*HI, G for *Bgl*II, D for *Dra*I, EI for *Eco*RI, EV for *Eco*RV, and X for *Xba*I.

To obtain genes encoding MYB-like transcription factors regulating the phenotypic difference between pink and variegated petals, 3′ rapid amplification of cDNA ends (RACE) PCR was performed for cDNA from pink petals using the G1709 and B25 primers (Fig. 5). Amplified fragments were cloned into the pGEM-T Easy cloning vector. cDNA clones derived from pink petals had various lengths of insert ranging from 860bp to 1020bp, but sequence similarity was very high between these clones. The amino acid sequence of the N-terminal region deduced from the pink clone cPpP14 showed high similarity to other plant MYB transcription factors. Among 21 R2-R3 MYB transcription factors, 33 out of 66 amino acids corresponding to the latter part of R2 and whole R3 repeat were identical (Fig. 8). Furthermore, 50 amino acids in the same region of cPpP14 were identical to *TT2*, *ZmC1*, *ZmPl*, and *OsC1*, whereas 37 were identical to *Rosea1*, *Venosa*, *An2*, *VvMYBA1*, and *PprMYB10*. However, the C-terminal region was different from other MYB proteins, which may reflect the specificity of this gene.

When this cPpP14 clone was used as a probe for hybridization to RT-PCR products on Southern blots to confirm the expression of this *MYB*-like gene in flower buds, the 1.0, 1.1, and 1.3kb transcripts were detected only in cDNA from pink flower buds (Fig. 4B).

Another *MYB*-like gene obtained from genomic DNAs of pink petals was almost identical to *PprMYB10*. The sequence of this gene, 1818bp in length, was confirmed by connecting overlapped clones. The *PprMYB10* gene is 1849bp in length. The deduced amino acid sequence of this gene started with the same first codon as *PprMYB10* and the sequence of this gene, including the 1st and 2nd introns, was identical to *PprMYB10*. Based on these results the *MYB10* gene from flowering peach was named as *FPMYB10*. Expression of *FPMYB10* in pink and variegated flower buds was investigated by RT-PCR Southern blots using two forward primers, A2F and B2F, in combination with the B25 reverse primer. The 3′ region of *FPMYB10*, amplified using C2F and C2R primers, was used as a probe. Pink and variegated flower buds showed strong and almost identical levels of expression of the *FPMYB10* cDNA amplified using either A2F and B2F primers (Fig. 4C). Therefore at least in petals, differences in expression of the *FPMYB10* gene are not the cause of the differences in colour between pink and variegated flowers.

### Function of the *MYB* gene obtained from pink petal cDNA

**Fig. 6.**
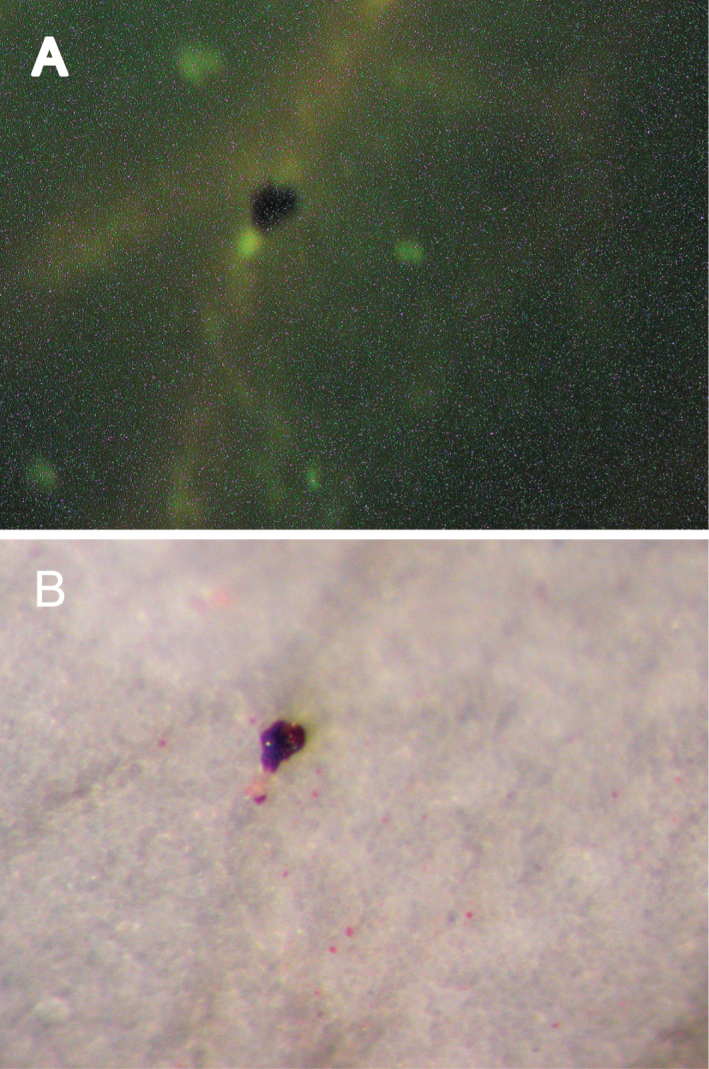
Complementation of pigment synthesis confirmed by particle bombardment into the white area of variegated petals with the *Peace* gene. (A) Yellow spots were generated by the *YFP* gene. (B) Magenta spots were generated by the *Peace* gene. Both observed in the same field of vision.

5′ rapid amplification of cDNA ends (RACE) PCR was performed to obtain the 5′ region of the cPpP14 cDNA clone using the gene-specific primer PGS42 based on the cPpP14 sequence (Fig. 5). The amplified fragment was cloned into the pGEM-T Easy vector and sequenced. The entire coding sequence of the flowering peach *MYB* gene was reconstructed from the sequence information of the 5′ RACE clone, cPpP33, carrying a 351bp fragment as an insert and 3′ RACE clone, cPpP14, having a 858bp insert. The full-length gene was then amplified from cDNA from pink petals with gene-specific primers; namely the PMintF primer designed just upstream of the start codon and the PMintR primer just 3′ to the first stop codon. The amplified product was diluted and used as a template for *att*B-PCR to add the *att*-B sequences, required for preparation of an entry vector for the GATEWAY cloning system (Life Technologies, USA). Amplified *att*-B product was inserted into the pDONR207 entry vector by BP recombination. The cDNA was transferred by LR recombination to the pJAM1500 vector where expression is driven by the double CaMV 35S promoter. The Peach *MYB* gene in this expression cassette was bombarded into variegated flower buds together with the *YFP* gene. Twenty-four hours later, bombarded petals were observed under the dissection microscope. Many magenta spots were observed surrounding aggregated gold particles (Fig. 6). The complementation ratio of 104.1% (Table 2) was much higher than the ratio achieved by bombardment with *Rosea1* (Table 1). It was concluded that this peach *MYB* gene was controlling the pigmentation of petals of flowering peach. Therefore, this gene was named *Peace* (*pe*ach *a*nthocyanin *c*olour *e*nhancement). In variegated flower petals, *Peace* expression was not detected by RT-PCR Southern hybridization when probed with clone cPpP14 (Fig. 4B). Consequently, the lack of *Peace* expression probably resulted in white areas in variegated petals.

**Table 2.** Magenta spot ratio acquired following introduction of the peach *MYB*-like gene (*Peace*) by particle bombardment

Transcription factors	Magenta spot/yellow spot (%)
Negative control	0.0
*YFP* control	0.0
Peach *MYB*-like gene (*Peace*)	104.1

### Comparison of *Peace* genomic and cDNA sequences between pink and variegated petals

The gene structure of *Peace* was confirmed by comparison of cDNA and genomic sequences. The *Peace* gene was composed of three exons disrupted by two introns, the first intron was 87bp in length located in the R2 domain at 133 from ATG and the second one was 80bp in length located in R3 domain at 261 from ATG in cDNA numbering (Fig. 5). These were identical positions to the introns in other plant *MYB* genes (Martin and Paz-Ares, 1997). Four candidate TATA box sequences were found within 100bp of the region upstream from the ATG, based on the TFBIND (Tsunoda and Takagi, 1999). One of these candidates, the TATA box situated at 53bp upstream of the initiating ATG codon, showed the highest score of 0.846 in TFBIND.

**Fig. 7.**
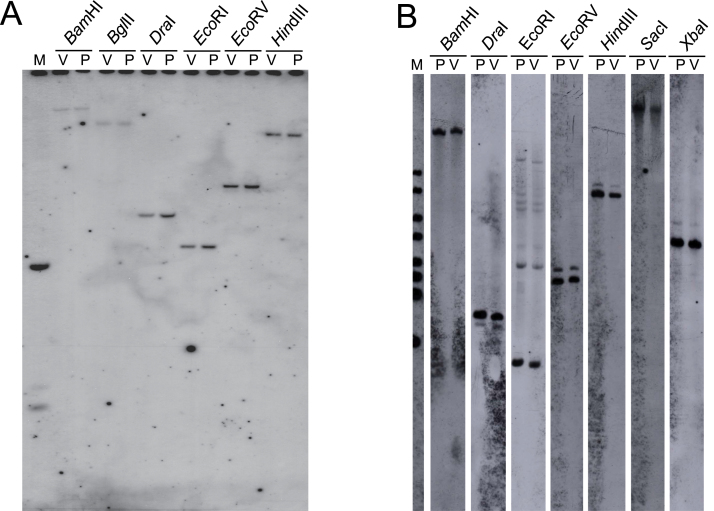
DNA gel blot of genomic DNA obtained from pink (P) and variegated (V) petals. (A) Probed with clone cPpP14, containing the 3′ end of the *Peace* gene. (B) Probed with clone cPpP3314, the full-length clone of the *Peace* gene.

To investigate the reasons why the *Peace* gene was strongly expressed in pink petals but not in variegated petals, a genomic library was constructed from V2 tissue and coding and 5′-UTR regions were compared between pink and variegated tissues. The genomic sequence of the coding region and *ca*. 3kb of promoter region of the *Peace* gene was determined by screening the V2 genomic library. Based on this sequence information, sequences of coding and promoter regions of the P2 genome was determined by PCR sequencing. The sequences of coding and promoter regions were completely identical in DNA from pink petals and from variegated petals. No polymorphisms were detected within coding and promoter regions. To confirm whether there was a difference between pink and variegated genomes, genomic Southern hybridization was performed under high stringency conditions using both the full-length cDNA clone of *Peace* (cPpP3314) and the cPpP14 clone containing just the 3′ region of *Peace* cDNA as probes. Both pink and variegated genomes carried a single copy of the *Peace* gene because hybridization patterns showed a single band for each digestion (Fig. 7A). Probes made from the cPpP14 clone were likely to be highly specific for the *Peace* gene because this part of the cDNA lacked most of the sequences encoding the MYB domain which is highly repeated in plants due to their large number of R2R3 MYB genes. Additional weaker signals were observed when the full-length cDNA clone (cPpP3314) was used as a probe (Fig. 7B). These weaker signals are probably due to hybridization to other genes encoding the conserved R2R3 MYB domain. However, no differences were observed between pink and variegated genomes which could explain the phenotypic differences in flower pigmentation.

To investigate another possible mechanism determining the difference in *Peace* expression between pink and variegated flowers, sites for DNA methylation were searched in the promoter region. Thirty-five CpG dinucleotides were found to be scattered in the promoter region, but there were no CpG islands in which CpG dinucleotides appeared at high density. Therefore, it seems unlikely that differential DNA methylation controls *Peace* gene expression in flowering peach.

### Relationship between Peace and other plant MYB transcription factors

**Fig. 8.**
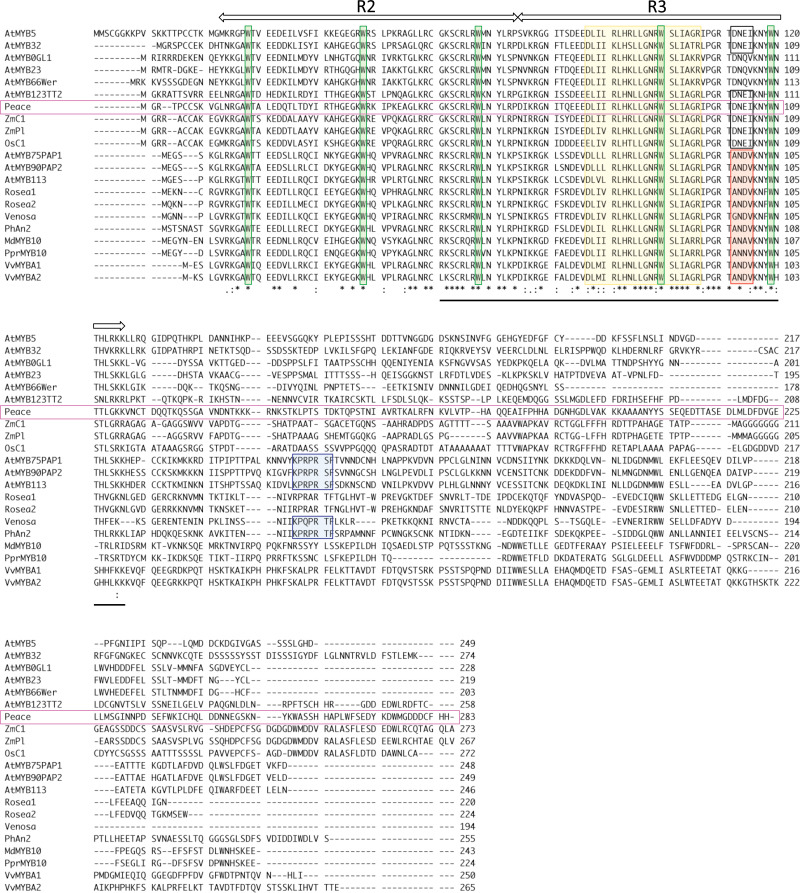
Alignment of the deduced amino acid sequences of 21 R2-R3 MYB transcription factors belonging to subgroup 4, 5, 6, 7, and 15. Peace is marked using pink. Conserved tryptophans are marked using green. The amino acid signature motifs of {[DE]Lx_2_[RK]x_3_Lx_6_Lx_3_R}, important for interaction with bHLH proteins, are indicated by a yellow square, the [A/S/G]N[D/A/N]V motif and the KPXPR[S/T]F motif, known to be conserved in anthocyanin regulators, are indicated by a red square and a blue square, respectively. The conserved amino acid sequence [DNEI] is shown by a black square. The horizontal line indicates the amino acid sequence of the R2-R3 region derived from the cPpP14 clone.

The amino acid sequence deduced from the full-length *Peace* cDNA revealed the protein to contain well-conserved R2 and R3 MYB domains. Peace aligned well with amino acid sequences of 20 MYB proteins belonging to subgroups 4, 5, 6, 7, and 15, chosen for their functions in regulating flavonoid biosynthesis or by close phylogenetic relationships to regulators of flavonoid metabolism from other plants (Fig. 8) (Stracke *et al.*, 2001; Bailey *et al.*, 2008; Dubos *et al.*, 2010). Among 22 MYB transcription factors, 45 deduced amino acids out of 104 in R2 and R3 repeats were identical. The region of highest conservation was the latter part of the R2 domain and middle and C-terminal part of the R3 domain. Five tryptophans, considered to be important to forming the two repeats of the helix-helix-turn-helix structure in the R2 and R3 domains, were completely conserved in Peace. Furthermore, Peace has the conserved amino acid signature motif [DE]Lx_2_[RK]x_3_Lx_6_Lx_3_R which is important for interaction with bHLH proteins from sub-group IIIf (Heim *et al.*, 2003; Zimmermann *et al.*, 2004; Lin-Wang *et al.*, 2010). These results suggest *Peace* can act to regulate anthocyanin biosynthesis in petals of flowering peach in combination with bHLH proteins. However, neither the [A/S/G]N[D/A/N]V motif (Lin-Wang *et al.*, 2010) nor the KPXPR[S/T]F motif, both known to be conserved in R2R3MYB proteins promoting anthocyanin biosynthesis, were present in Peace. The C-terminal region of Peace was quite divergent compared with those of other plant MYB proteins.

**Fig. 9.**
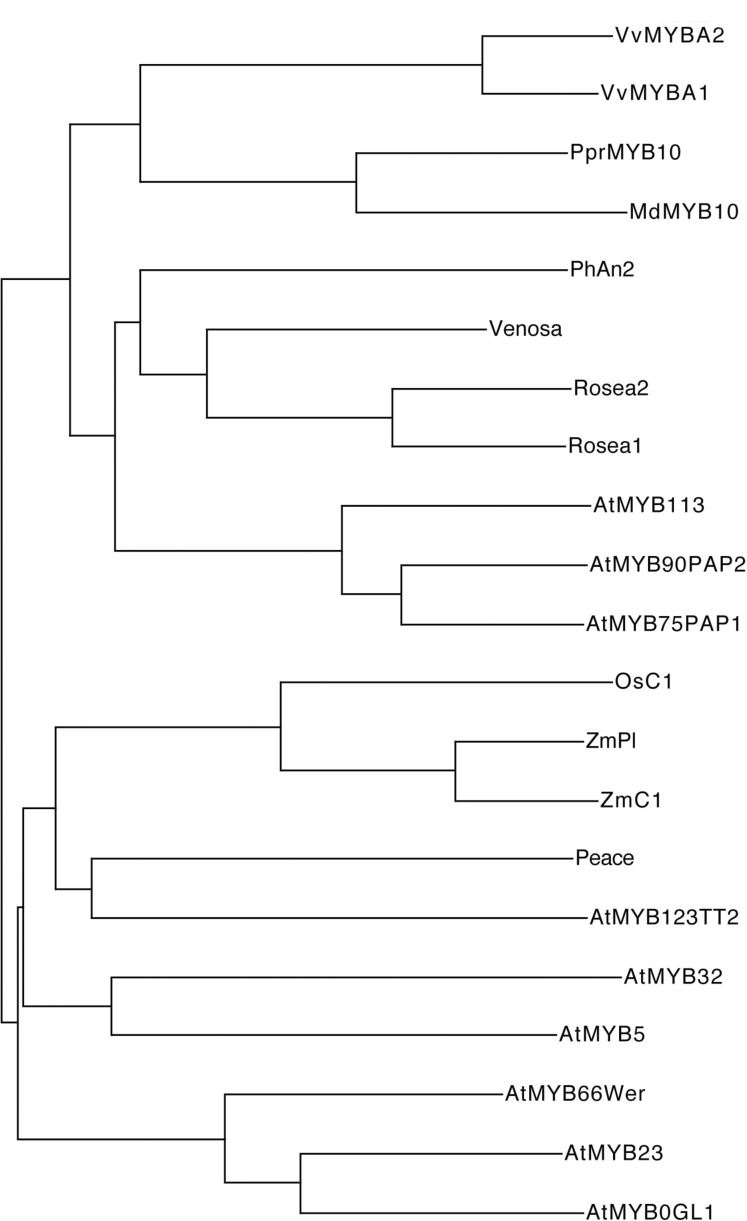
Phylogenetic tree of R2R3 MYB transcription factors showing the relationship between Peace and another 20 accessions belonging to subgroups 4, 5, 6, 7, and 15; the same as shown in Fig. 8.

To compare the amino acid sequences of Peace, phylogenetic analysis was undertaken for it and 20 additional MYB transcription factors (Fig. 9). The phylogenetic tree indicated that Peace was most closely related to AtMYB123 (TT2) of *Arabidopsis* which regulates proanthocyanidin biosynthesis (Nesi *et al.*, 2001; Baudry *et al.*, 2004). Monocotyledonous MYB proteins, C1 and Pl of *Zea mays* and OsC1 of *Oryza sativa*, involved in the anthocyanin biosynthesis (Paz-Ares *et al.*, 1987; Cone *et al.*, 1993) were also closely related to Peace. However, PprMYB10 of *Prunus persica* (peach) was not closely related to Peace. It was clustered together with MdMYB10, VvMYBA1, and VvMYBA2 (Kobayashi *et al.*, 2004).

## Discussion

The *Peace* gene encoding an R2R3 MYB-like transcription factor that is expressed in pink petals of flowering peach, *Prunus persica* cv. Genpei which bears pink flowers and variegated flowers on a single tree has been identified. This gene is strongly expressed in fully pigmented pink petals but very weakly expressed in variegated petals. Introduction of the *Peace* gene into the white areas of variegated petals by means of particle bombardment complemented pigment biosynthesis and magenta spots appeared. It was, therefore, concluded that the *Peace* gene is likely to be responsible for colouration in the petals of flowering peach.

All structural genes involved in the anthocyanin biosynthetic pathway were strongly expressed in young petals of pink flower buds, whereas *CHI*, *F3H*, and *ANS* genes showed only weak expression and no expression of *PAL*, *CHS*, and *DFR* genes were observed in variegated flower buds. These results agreed well with the result of pigment analysis which showed that pink petals contained >100-fold more cyanidin-3-glucoside than variegated petals. It seems likely that a change affecting expression of all the anthocyanin biosynthetic genes has occurred in a gene encoding a transcription factor regulating their expression. In this context, the expression pattern of *Peace*, which is strongly expressed in pink petals but not expressed in variegated petals, supports the view that Peace regulates the anthocyanin biosynthetic pathway and that it is variation in its expression that gives variegated petal pigmentation in flowering peach petals.

When Peace was compared with 20 other MYB transcription factors from plants, high similarity was seen between the 104 amino acids of the R2R3 domain, including the identity in some key amino acid positions which are important for interaction with bHLH proteins. This conservation also suggests that *Peace* encodes a transcription factor controlling anthocyanin biosynthesis in the petals of flowering peach, although a gene encoding a bHLH protein partner has not yet been identified.

Peace does not have the [A/S/G]N[D/A/N]V motif, which appears near the end of the R3 repeat and can be an identifier for anthocyanin regulators (Lin-Wang *et al.*, 2010). However, at the same position, Peace shares the amino acid sequence DNEI with monocotyledonous anthocyanin regulators; ZmC1, ZmPl, and OsC1, and with AtMYB5, AtMYB32, and AtMYB123 (TT2). This suggests that Peace is functionally more similar to AtMYB123 and AtMYB5 which are known to regulate proanthocyanidin production partially redundantly and to monocotyledonous anthocyanin regulators such as ZmC1, ZmPl or OsC1 (Gonzalez *et al.*, 2009; Dubos *et al.*, 2010). VvMYB5a, the protein from grapevine most similar to AtMYB5 has been shown to activate anthocyanin biosynthesis when expressed ectopically in tobacco (Deluc *et al.*, 2006).

Phylogenetic analysis showed that Peace clustered with AtMYB123 and anthocyanin regulators of monocotyledonous plants, such as ZmC1, ZmPl, and OsC1. Stracke *et al.* (2001) showed a correlation between functional conservation and sequence similarity in MYB transcription factors, for example, AtMYB123 and ZmC1 both regulate pigmentation. Therefore it seems possible that Peace has retained the capacity to regulate anthocyanin biosynthesis by a similar manner as the regulators of anthocyanin biosynthesis from monocots or it has arisen *de novo* from a proanthocyanidin regulatory ancestor. It may be that the regulatory activity of Peace reflects the ability of other regulators of proanthocyanidin biosynthesis from dicots to regulate anthocyanidin biosynthesis, a function not previously recognized because of their concomitant activation of ANR and proanthocyanidin production. Recently, Ravaglia *et al.* (2013) suggested that MYB10 and MYBPA1 of nectarine (*Prunus persica*) exclusively regulate anthocyanin biosynthesis and proanthocyanidin biosynthesis, respectively, in the fruit skin and flesh. However, genes similar to Peace may also contribute to regulating anthocyanin biosynthesis and proanthocyanidin biosynthesis in peach.

The mechanisms regulating the difference in the expression of the *Peace* gene between pink and variegated petals remains unclear. *FPMYB10*, a *PprMYB10* homologue, was a possible candidate to control the difference in flower colour or the difference in *Peace* gene expression. However, both in pink and variegated flower buds, *FPMYB10* is expressed at same level (Fig. 4C) and therefore it was difficult to consider this gene to be the cause of the difference in flower colour or *Peace* expression between pink and variegated flowers.

Another possible mechanism regulating the difference in Peace expression, could involve miRNA or siRNA. These are known to regulate expression of transcription factors including MYB proteins (Rhoades *et al.*, 2002; Allen *et al.*, 2007). Furthermore SERRATE in *Arabidopsis* could affect miRNA function to regulate gene expression (Lobbes *et al.*, 2006). Recently, miR156-targeted SPL (SQUAMOSA PROMOTER BINDING PROTEIN-LIKE) gene was revealed negatively regulating anthocyanin biosynthesis in *Arabidopsis* by disrupting the MYB-bHLH-WD40 complex (Gou *et al.*, 2011). In order to understand the mechanism regulating the difference in Peace expression, further investigations are required.

Both pink and variegated genomes possess a single copy of the *Peace* gene, according to the results of genomic Southern hybridization. Sequence analysis, in addition to genomic Southern blots, suggests the *Peace* gene to be identical in pink and variegated flowers, refuting previous suggestions that variegation results from insertion of a transposable element (Chaparro *et al.*, 1995). It is possible that the variegated phenotype in flowering peach cv. Genpei results from a periclinal chimera, where the L1 has been derived from a colourless genotype and the L2/L3 from a pink genotpoye. Pink sectors on flowers and branches with pink flowers could then result from L2 layer invasion, as observed in some grape varieties (Pelsy, 2010). However, no evidence for a chimera was observed in analysis of *Peace* by Southern blot of DNA from pink and variegated flowers. In addition, variegated plants of weeping flowering peach breed true to give progeny with variegated flowers, suggesting that variegation is not due to variegated flowering peach being a periclinal chimera (H Katayama, unpublished observations).

Although the *Peace* gene, whose expression controls the pigmentation of flowers in flowering peach, has been identified, the molecular basis for the variegation in flower colouration remains a mystery.
